# Early outcomes of robotic versus laparoscopic splenectomy in pediatric population: a systematic review and meta-analysis

**DOI:** 10.1186/s12887-025-06198-z

**Published:** 2025-10-07

**Authors:** Nada Osama Aboelmajd, Moaz Yasser Darwish, Mariam Ahmed Orabi, Abanoub Gamil Abdelmalek Ghabious, Taha Abd-ElSalam Ashraf Taha, Nada K. Abdelsattar

**Affiliations:** 1 Faculty of Medicine, South Valley University, Qena, Egypt; 2 Faculty of Medicine, Fayoum University, Fayoum, Egypt; 3 Faculty of Medicine, Cairo university, Cairo, Egypt

**Keywords:** Robotic, Laparoscopic, Splenectomy, Pediatric surgery

## Abstract

**Background:**

Laparoscopic splenectomy (LS) is currently well-established as a standard technique for splenectomy. Robotic splenectomy (RS) is being introduced as a new minimally invasive alternative. The current study aims to directly compare RS and LS specifically in the pediatric population undergoing splenectomy for non-traumatic indications.

**Methods:**

We performed a systematic search in four databases: PubMed, Web of Science, Scopus and Cochrane CENTRAL in November 2024. We included studies that compared outcomes of RS and LS in pediatric patients. We extracted the amount of blood loss, operation time, length of hospital stay, the number of patients who needed blood transfusion or were converted to open approach and post-operative complications. Finally, RevMan software was adopted for meta-analysis.

**Results:**

Six retrospective studies met our eligibility criteria and were included in the current systematic review and meta-analysis with a total of 248 patients, of which 123 and 125 children underwent RS and LS respectively. Blood loss was significantly lower in RS group (MD = -56.95, *P* = 0.01). Operation time, hospital stay, the need for blood transfusion and post-operative complications showed no significant difference between both RS and LS groups. Despite the overall good quality of the included studies, the GRADE quality of evidence was considered very low due to the observational nature of the included studies, small sample sizes and high variability between outcomes of different studies.

**Conclusion:**

Our study guides the existing literature towards a possible non-inferior status of robotic splenectomy compared to the well-established laparoscopic splenectomy in most clinical outcomes. Blood loss was significantly lower in children who underwent robotic splenectomy, but small sample size limits generatability of such finding. The challenge of higher costs of robotic splenectomy needs to be addressed in well-designed cost-effectiveness studies.

**Supplementary Information:**

The online version contains supplementary material available at 10.1186/s12887-025-06198-z.

## Introduction

Splenectomy is a well-established surgical intervention in pediatric population. It is frequently indicated for hematologic conditions, hypersplenism and splenic tumors [1, 2]. Laparoscopic splenectomy (LS) is known as the gold standard for such patients when feasible, providing excellent outcomes in experienced hands [3, 4]. However, technical challenges encountered during the procedure, which may result in prolonged operative duration and elevated complication rates, underscore the necessity to identify a new minimally invasive alternative [5].

Robotic-assisted splenectomy (RS) is a potential alternative procedure which may shorten operative time, minimize hospital stay and reduce blood loss. The wristed instruments, along with 3D magnification, provide enhanced visualization and motion control with greater precision [1]. However, a previously published meta-analysis reported the absence of a significant difference between RS and LS in terms of operative time and hospital stay in adult population [6]. Additionally, the cost-effectiveness of RS has been questioned, taking into consideration the prolonged setup time, which is particularly relevant in the context of pediatric surgery, where resource allocation and procedural efficiency are critical factors [7].

Many studies investigated the use of RS for hematological indications in adult population [6, 8–10]. However, the use of robotic surgery in pediatric population has been limited due to technical difficulties, including instruments size and general lack of experience. Recently, there has been an increase in research reports on the use of RS in pediatric patients, highlighting the need for a comprehensive evidence synthesis tailored to this age group [1, 2, 7, 11–13].

The current research work is a systematic review that aims to offer robust evidence about the role of RS in pediatric population. Incorporating meta-analysis models intends to provide direct comparisons between RS and LS in terms of key peri-operative outcomes, particularly operation time, conversion to open surgery, postoperative complications, length of hospital stay, blood transfusion and blood loss.

## Methods

We performed a systematic review and meta-analysis following the preferred reporting items for systematic review and meta-analysis (PRISMA) in accordance with the guidelines of Cochrane handbook [14, 15].

### Eligibility criteria

Our systematic review included English language studies that compared laparoscopic and robotic approaches of splenectomy in children under 18 years old. Case-reports and conference abstracts were excluded. There were no further restrictions regarding study design or date of publication.

### Database search

In November 2024, we performed a systematic search in four databases: PubMed, Web of Science, Scopus and Cochrane CENTRAL. We searched the literature using the search strategy: (laparoscopy OR laparoscopic OR laparoscopes OR laparoscope OR Laparoscopic OR laparoscopically OR laparoscopically OR laparoscopy OR robotic OR “robot-assisted” OR “robot assisted” OR robotically OR robotics OR robotization OR robotized OR robots) AND (paediatrics OR pediatrics OR paediatric OR pediatric OR children)) AND (splenectomy OR splenectomies OR spleen resection).

### Study identification

Identified records were imported into EndNote in order to remove duplicates. Subsequently, records were exported to Excel, where titles and abstracts were examined by two independent reviewers. Finally, full-text screening was performed in accordance with the previously mentioned eligibility criteria. Any disagreement was resolved by a third reviewer.

### Data extraction

General information, baseline characteristics, safety and efficacy outcomes were extracted from each study by two independent reviewers and revised by a third one. General information included: study ID, study design, total sample size, sample size for each arm, indication of splenectomy and follow-up period after splenectomy. Baseline data included: age, sex, BMI, hematological diseases, spleen weight, and spleen longitudinal diameter for each study arm. Efficacy and safety outcomes included operation time, blood loss, postoperative complications, need for blood transfusion, length of hospital stay, conversion to open surgery.

### Risk of bias

The Risk Of Bias In Non-randomized Studies - of Interventions (ROBINS-I) tool was adopted to assess the quality of the included non-randomized retrospective cohort studies [16]. The seven quality domains were assessed for each study by two independent reviewers and a third reviewer handled disagreements. We implemented the GRADE approach system in order to estimate the quality of evidence; two reviewers adopted the Grading recommendations assessment, which categorizes each outcome into one of four levels: high, moderate, low, or very low [17].

### Statistical analysis

Meta-Analysis was performed using Review Manager (RevMan) software version 5.4. Regarding dichotomous outcomes, pooled risk ratios (RRs) with 95% confidence interval (CI) were calculated. Mean difference (MDs) with 95% CIs were calculated for the continuous outcomes. Following the guidelines of Cochrane handbook for systematic reviews of interventions, (2) Heterogeneity was assessed using Chi-Square and I-Square tests. Whenever the p-value of Chi-Square test was < 0.1 or I-Square test result was > 50% in a meta-analysis model, studies were considered heterogeneous. A random-effect model was adopted for heterogenous studies; otherwise, a fixed-effect model was used. Additionally, whenever heterogeneity was observed, a sensitivity analysis was performed by removing one study in each scenario.

## Results

### Data collection and study selection

**Fig. 1:**
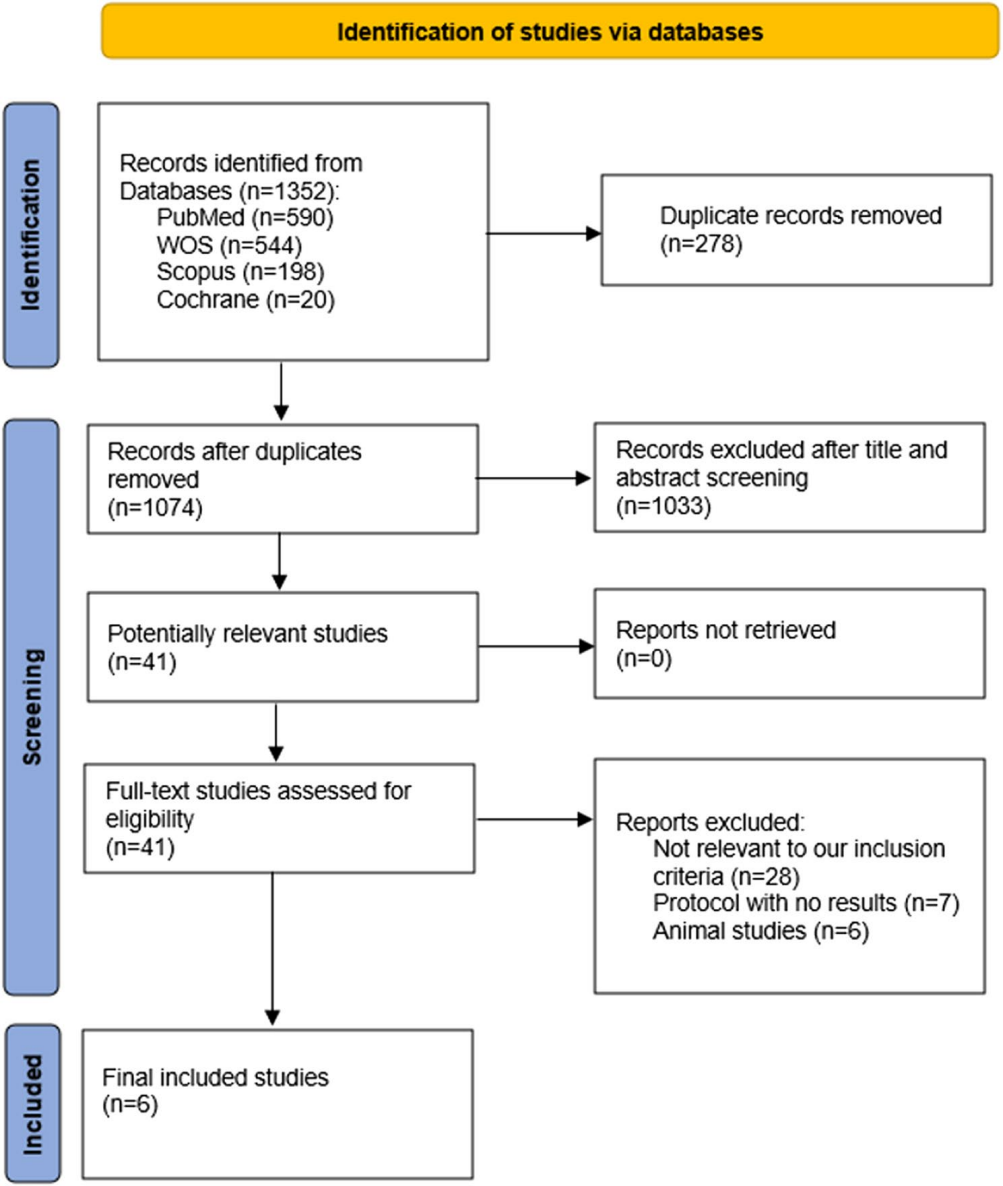
PRISMA flow diagram of the included studies

Our systematic search yielded 1,352 records from different databases (PubMed = 590, WOS = 544, Scopus = 198 and Cochrane = 20), of which 278 duplicated records were identified and removed. Subsequently, 1,074 unique records were examined for eligibility through title and abstract screening. Of these, 41 records were eligible for full-text screening, and ultimately, only 6 studies fulfilled the eligibility criteria and were included in evidence synthesis. The study selection process is illustrated in Fig. 1.

### Characteristics of the included studies

All of the six included studies were retrospective cohort studies with a total population of 248 children. RS and LS were the adopted interventions in 123 and 125 children, respectively. Indications of splenectomy varied between studies, including different hematological causes such as sickle cell disease, thalassemia and recurrent need of blood transfusion. Additional details regarding general information and baseline characteristics are provided in Tables 1 and 2.

**Table 1:** General characteristics of included studies

Study ID	Study design	Country	Total sample size	Laparoscopic group sample size	Robotic group sample size	Indications of Splenectomy	Follow up time
Belharbi 2023 [11]	Retrospective cohort	France	41	26	15	Benign homeopathies [N=36]: (sickle cell disease, thalassemia, pyruvate kinase deficiency, Blackfan-Diamond, Minkowski-Chauffard, idiopathic thrombocytopenic purpura), for tumors [N=15]: (epithelial, mesothelial, dermoid), and for a case of Nieman Pick disease [N=1].	-
Cai 2023 [12]	Retrospective cohort	China	32	19	13	-	-
Delgado‐Miguel 2024 [2]	Retrospective cohort	-	84	23	61	Splenectomy was moderate to severe anemia and recurrent blood transfusions (>2 per year).	-
Shelby 2021 [13]	Retrospective cohort	USA	24	14	10	-	-
Vasilescu 2012 [7]	Retrospective cohort	-	32	22	10	Severe anemia and recurrent need of blood transfusion in all patients.	4-103m
Zhang 2024 [1]	Retrospective cohort	-	35	21	14	-	2-28m in Robotic group, 3-12 m in Laparoscopic

**Table 2:** Baseline characteristics of included studies

Study ID	Study groups	Age	Males	BMI	Hematological disease N (%)			Spleen weight	Spleen longitudinal diameter
		Mean ± SD	N (%)	Mean ± SD	Hereditary spherocytosis	Sickle cell	ITP	Mean ± SD	Mean ± SD
Belharbi 2023 [11]	Laparoscopic	11 ± 1.625	-	-	-	-	-	-	-
	Robotic		-	-	-	-	-	-	-
Cai 2023 [12]	Laparoscopic	11.14 ±3.7	11(61.1)	-	-	-	-	-	-
	Robotic	12.37 ±5.14	5(50)	-	-	-	-	-	-
Delgado‐Miguel 2024 [2]	Laparoscopic	9.7±9.1	11(47,8)	21.2±5.5	6(26.1)	13(56.5)	3(13)	-	14.8±3.7
	Robotic	8.7±4,9	37(60.7)	19.8±4.7	15(24.5)	43(70.5)	1(1.6)	-	15.4±3.6
Shelby 2021 [13]	Laparoscopic	11.3± 6.3	8(57)	-	0 (0%)	12 (86%)	2 (14%)	-	Spleen size:12.2±2.4
	Robotic	9.9 ±4.5	6(60)	-	6 (60%)	2 (20%)	2 (20%)	-	14.5 ± 2.1
Vasilescu 2012 [7]	-	-	-	-	-	-	-	-	-
Zhang 2024 [1]	Laparoscopic	-	9(42.80	-	-	-	-	-	-
	Robotic	-	7(50)	-	-	-	-	-	-

### Outcomes

#### Blood loss

**Fig. 2:**
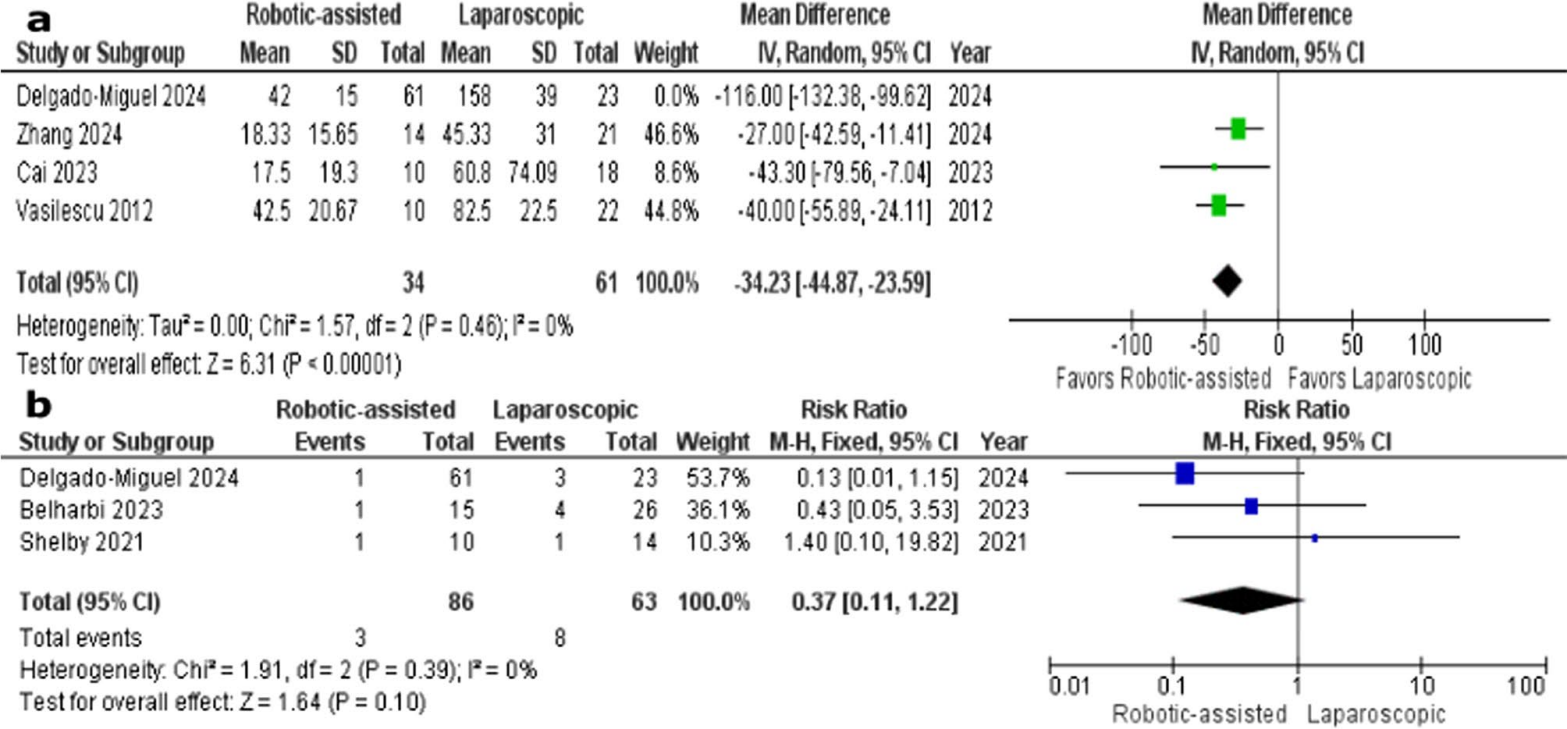
**a:** Sensitivity analysis of the amount of blood loss, **b**: Forest plot of the number of patients who needed blood transfusion

The amount of blood loss was reported in four studies [1, 2, 7, 12]; RS group included 95 patients and LS group included 84 patients. The amount of blood loss was significantly lower in RS group (MD = −56.95, 95% CI, −101.59 to −12.30, *P* = 0.01). Heterogeneity was observed between studies (*P* < 0.00001, I^2^ = 96%). Heterogeneity was best resolved by excluding the study of Delgado-Miguel 2023 (P 0.46, I^2^ = 46%). After omitting Delgado-Miguel 2023 from the meta-analysis model, the overall mean difference still favored RS group over LS group (MD = −34.23; 95% CI, −44.87 to −23.59; *P* < 0.00001) (Fig. 2A).

#### Blood transfusion

The incidence of blood transfusion was reported in three studies [2, 11, 13]; RS group included 86 patients and LS group included 63 patients. The overall risk ratio did not favor either RS or LS group (RR = 0.37; 95% CI, 0.11 to 1.22; *P* = 0.10). The included studies showed homogeneity (*P* = 0.39, I^2^ = 0%) (Fig. 2B).

#### Operation time

**Fig. 3:**
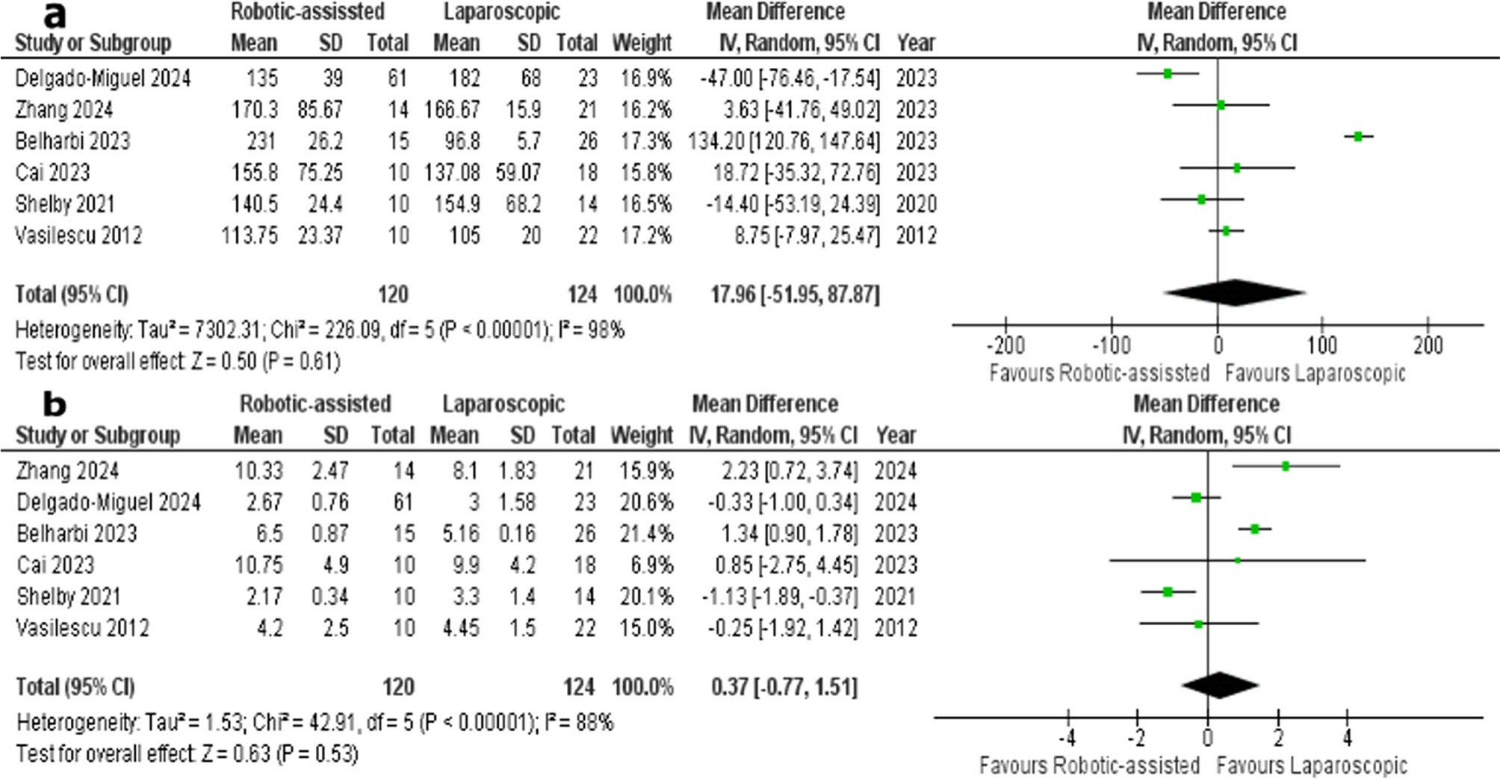
**a:** Forest plot of operation time, **b**: Forest plot of length of hospital stay

Six Studies reported operation time for both RS and LS groups [1, 2, 7, 11–13]; RS group included 120 patients and LS group included 124 patients. The overall mean difference did not favor either RS or LS groups (MD = 17.96; 95% CI, −51.95 to 87.87; *P* = 0.61). The included studies showed heterogeneity (*P* < 0.00001, I^2^ = 98%) indicating variability across studies. Removing one study in each scenario did not resolve heterogeneity (Fig. 3A).

#### Length of hospital stay

Length of hospital stay was reported in six studies [1, 2, 7, 11–13]; RS group included 120 patients and LS group included 124 patients. The overall mean difference did not favor either of the two groups (MD = 0.37; 95% CI −0.77 to 1.51; *P* = 0.53). Heterogeneity was observed between the included studies (*P* < 0.00001, I^2^ = 88%). Heterogeneity remained unresolved after removing single study in each scenario (Fig. 3B)

#### Post-operative complications

**Fig. 4:**
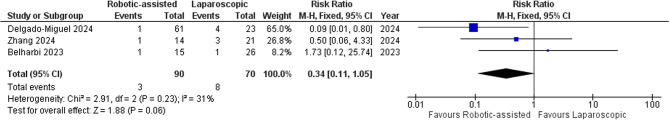
Forest plot of pot-operative complications

The outcome of postoperative complications was reported in three studies [1, 2, 11]; RS group included 90 patients and LS group included 70 patients. The overall risk ratio did not favor either of the two groups (RR = 0.34; 95% CI, 0.11 to 1.05; *P* = 0.06). The included studies were homogenous (*P* = 0.23, I^2^ = 31%) (Fig. 4).

### Quality assessment

Using the GRADE methodology, we determined that the overall quality of evidence supporting our findings was very low. A detailed breakdown of evidence quality, effect magnitudes, and risk estimation sources is provided in Supplementary Table S1 [Additional file 1].

### Risk of bias

**Fig. 5:**
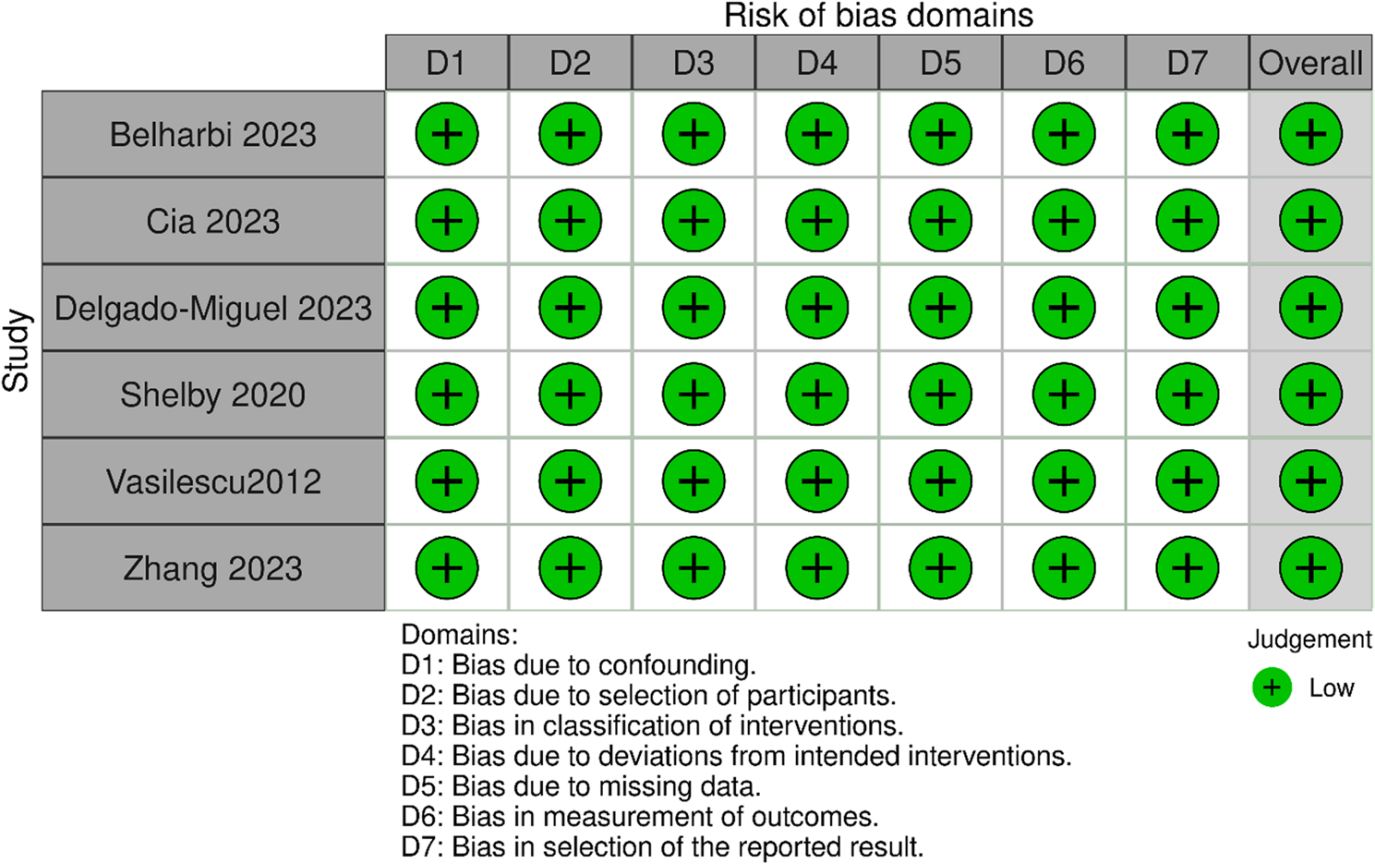
Quality assessment of the included studies using ROBINS-I tool

Our assessment using the ROBINS-I tool revealed low risk in all of the included studies, this includes low risk of bias in all of the domains; Participants selection, intervention classification and outcome measurement. The quality assessment figure is found in Fig. 5.

## Discussion

Robotic surgical techniques are increasingly gaining the interest of surgeons across various specialties due to their potential to overcome the shortcomings of the well-recognized laparoscopic techniques [18]. Despite being well-established, LS still poses some challenges, such as the required meticulous dissection of the spleen and its vascular pedicle to control intra-operative bleeding loss [19]. RS, with its magnification abilities, has been explored lately in the literature as a potential alternative to LS [6, 20].

The anatomical specifications of the pediatric spleen along with technical challenges related to RS make it important to investigate the outcomes of such procedure in the pediatric age group specifically [21]. Our systematic review and meta-analysis aim to review the current state of evidence about RS in pediatric patients. It provides direct comparisons between RS and LS to evaluate the advantages of each approach over the other in this specific age group.

Blood loss is a critical intraoperative complication, especially in children, where hemorrhage disturbs hemodynamic stability quickly compared to adults [11]. In our study, blood loss was significantly lower among pediatric patients in RS group compared to LS group. The meta-analysis of Peng et al. reviewed and analyzed the literature of RS in adult population and found a significant reduction in blood loss which goes in alignment with our findings in pediatric population (*P* = 0.0006) [6].

Intraoperative blood loss was previously reported as the principal indication for conversion of laparoscopic splenectomy to open approach, as bleeding could obscure surgeon’s view [22, 23]. Additionally, splenomegaly could increase the risk of such blood loss, which is the case in many hematological indications of splenectomy [24, 25]. The enhanced precision and motion control of the robotic technique may be responsible for the lower amounts of blood loss, especially for the non-traumatic indications, which is the case in both the current study and the previous published one on adult population [6].

Despite the significant difference in terms of amount of blood loss, the rate of patients who needed blood transfusion showed no significant difference between RS and LS groups. This could be attributed to the limited number of studies that reported the outcome, as only 3 studies provided blood transfusion data [2, 11, 13]. More importantly, blood transfusion is a procedure governed by strict guidelines; therefore, not all cases of blood loss are indicated for blood transfusion [26]. Additionally, the need for blood transfusion is influenced not only by the severity of blood loss but also by the patient’s baseline condition.

Meta-analysis of conversion to open approach was not feasible in our study, as one of the three studies that included this outcome reported no events of conversion in both groups [2]. Overall, the three studies reported 2 conversion events out of 81 patients in the RS group, compared to 4 events out of 55 patients in the LS group [2, 12, 13]. The Ghidini et al. meta-analysis that was published in 2022 reported no significant difference in terms of conversion rate between RS and LS groups in pediatric population (*P* = 0.3) [20].

On the other hand, Peng et al. reported a significantly lower conversion rate in RS group among adult population (*P* = 0.02) [6]. This discrepancy in findings between adult and pediatric populations could be attributed to the learning curve associated with the robotic approach in pediatric patients, as the introduction of RS in adults began earlier than in the pediatric population.

In terms of operation time, our study found no significant difference between RS and LS groups in pediatric patients. This finding aligns with the results of Peng et al., where they found no significant difference in adult population as well (*P* = 0.72) [6]. This insignificant difference might be related to the relatively limited experience of surgeons in the field of robotic surgery compared to laparoscopic techniques. Additionally, the length of hospital stay showed no significant difference between groups in both pediatric population of our study (*P* = 0.53) and adult population of the Peng et al. meta-analysis (*P* = 0.39) [20].

The long operation time was one of the initial challenges encountered during the introduction of LS to the surgical community as an alternative to open surgery [27–29]. However, as surgeons gained experience with the technique, the difference in operative time between LS and the open approach has arrived at insignificant levels in some reports [19, 30, 31]. Based on this earlier experience, it may be reasonable to suggest a potential decrease in operation time for RS with continued practice and familiarity.

Our study found no significant difference between RS and LS groups in terms of post-operative complications, which goes in alignment with the results of Ghidini et al. in pediatric population (*P* = 0.235) [20]. On the other hand, the meta-analysis of Peng et al. reported a significantly lower rate of post-operative complications in RS group (*P* = 0.05) [6]. RS is indeed characterized by minimal tissue manipulation and high precision. However, the unique anatomical considerations of the pediatric spleen, along with the limited experience of surgeons in pediatric cases, may help explain the lower complication rates in adult RS [6, 21].

The literature status did not offer sufficient data suitable for meta-analysis of RS cost compared to LS. Delgado-Miguel et al. 2024, one of the retrospective studies included in our systematic review, reported no significant difference in median economic costs among 84 pediatric patients (*P* = 0.215) [2]. These findings align with a previously published review by Bhat et al. in 2022 that concluded the absence of statistically significant difference between RS and LS in adult population (*P* = 0.74). However, the cost analysis of Bhat et al. was based on two included studies only, which limits reliability and generalizability of the findings [32]. These results guide the scientific community toward a potential non-inferior status of RS compared to LS in terms of cost-effectiveness across both adult and pediatric populations. Nonetheless, the evidence regarding cost-effectiveness is considered limited with small number of studies and small population samples.

The potentially high cost associated with robotic techniques remains one of the major barriers to their widespread adoption. Therefore, cost-effectiveness studies are needed to enrich the current literature and provide a more comprehensive evaluation of such innovative approach, directing the surgical community toward the next step in the field. Additionally, the higher costs of the robotic technique restrict its availability in regions with scarce resources, which limits generalizability of the results. Multi-center cost-effectiveness studies with sufficiently large samples are necessary to establish solid evidence regarding RS cost compared to LS.

Our systematic review and meta-analysis comprehensively reviewed the current state of evidence regarding robotic techniques of splenectomy in the pediatric population. This study investigated the outcomes of RS in an age group where splenectomy is widely indicated due to various non-traumatic causes, especially the hematological ones.

Heterogeneity was high in 3 out of 5 meta-analysis models in our study (blood loss, operation time and length of hospital stay). Random-effect model was adopted in these models and sensitivity analysis was conducted as well. However, heterogeneity was only resolved in the meta-analysis models of blood loss. Additionally, the relatively scarce literature limited our ability to perform meta-regression or subgroup analyses based on child age or spleen size in order to explore sources of heterogeneity.

Another limitation is the exclusive retrospective design of the available literature that compares the outcomes of RS and LS. Despite being the basis for future plans for prospective studies, retrospective studies are prone to selection and recall biases as well as missing information. Since retrospective studies were not originally designed to collect data for research purposes, prospective studies are necessary to overcome the drawbacks of the current evidence.

The observational nature of the included studies had a major contribution to the very low GRADE quality of evidence. In addition, unresolved heterogeneity of operation time and hospital stay outcomes, and the wide confidence interval of all outcomes except hospital stay. The wide and imprecise confidence intervals are probably attributed to small sample sizes and high variability of study outcomes [33]. Overall, well-designed, prospective, multicenter clinical trials with larger populations and considerable quality are needed in the future.

In conclusion, our study guides the existing literature towards a possible non-inferior status of robotic splenectomy compared to the well-established laparoscopic splenectomy in most clinical outcomes. Blood loss was significantly lower in children who underwent robotic splenectomy, but small sample size limits generatability of such finding. The challenge of higher costs of robotic splenectomy needs to be addressed in well-designed cost-effectiveness studies.

## Supplementary Information

Supplementary Material 1.

## Data Availability

Data that support the findings presented in this manuscript will be made available upon reasonable request.
