# Supplementary material for: Early outcomes of robotic versus laparoscopic splenectomy in pediatric population: a systematic review and meta-analysis
Source: BMC Pediatr. 2025 Oct 7;25:781. doi: 10.1186/s12887-025-06198-z (PMC12502444; doi:10.1186/s12887-025-06198-z)
Supplement: Supplementary file 1 — Supplementary Material 1. [file 12887_2025_6198_MOESM1_ESM.docx]

**Early outcomes of robotic versus laparoscopic splenectomy in pediatric population: a systematic review and meta-analysis**

Authors: Nada Osama Aboelmajd^1^, Moaz Yasser Darwish^2^, Mariam Ahmed Orabi^3^, Abanoub Gamil Abdelmalek Ghabious^4^, Taha Abd-ElSalam Ashraf Taha^5^ and Nada K. Abdelsattar^6^

1. Faculty of Medicine, South Valley University, Qena, Egypt

[nada.osama@med.svu.edu.eg](mailto:nada.osama@med.svu.edu.eg)

1. Faculty of Medicine, Fayoum University, Fayoum Egypt

[my1336@fayoum.edu.eg](mailto:my1336@fayoum.edu.eg)

1. Faculty of Medicine, South Valley University, Qena, Egypt

[mariamoraby56@gmail.com](mailto:mariamoraby56@gmail.com)

1. Faculty of Medicine, Cairo university, Cairo, Egypt

[abanoub-abdelmalak@students.kasralainy.edu.eg](mailto:abanoub-abdelmalak@students.kasralainy.edu.eg)

1. Faculty of Medicine, Fayoum University, Fayoum Egypt

[ta1295@fayoum.edu.eg](mailto:ta1295@fayoum.edu.eg)

1. Faculty of Medicine, Fayoum University, Fayoum Egypt

[nk1267@fayoum.edu.eg](mailto:nk1267@fayoum.edu.eg)

***Corresponding author:**

Moaz Yasser Darwish

[my1336@fayoum.edu.eg](mailto:my1336@fayoum.edu.eg)

The GRADE quality assessment approach indicated that the quality of our evidence-based results is Very low. Table S1 shows a summary of the evidence's quality, the degree of the effect, and the source of information used in the estimated risk.

**Table S1. The quality of evidence as assessed by GRADE approach**

| **Certainty assessment** | | | | | | | **Certainty** |
| --- | --- | --- | --- | --- | --- | --- | --- |
| **No of studies** | **Study design** | **Risk of bias** | **Inconsistency** | **Indirectness** | **Imprecision** | **Other consideration** |  |
| **Operative time** | | | | | | | |
| 6 | Retrospective cohort | Not serious | Very serious ^a^ | Not serious | Serious^b^ | None | ⨁ Very low |
| **Postoperative complications:** | | | | | | | |
| 3 | Retrospective cohort | Not serious | Not Serious | Not serious | Serious^c^ | None | ⨁ Very low |
| **Length of Hospital stay:** | | | | | | | |
| 6 | Retrospective cohort | Not serious | Very Serious^d^ | Not serious | Not Serious | None | ⨁ Very Low |
| **Blood transfusion** | | | | | | | |
| 3 | Retrospective cohort | Not serious | Not Serious | Not serious | Serious^e^ | None | ⨁ Very Low |
| **Blood loss:** | | | | | | | |
| 4 | Retrospective cohort | Not serious | Not seriousᶠ | Not serious | Seriousᵍ | None | ⨁ Very low |

**CI:** confidence interval

**I^2^**: inconsistency index (I^2^)

**Explanations**

1. Might represent considerable heterogeneity, , I^2^=98%, sensitivity analysis did not resolve heterogeneity.
2. Wide CI, MD 17.96, 95% CI [-51.95, 87.87]
3. Wide CI, RR 0.34, 95% CI [0.11, 1.05]
4. Might represent substantial heterogeneity, I^2^=88%, sensitivity analysis did not resolve heterogeneity.
5. Wide CI, RR 0.37, 95% CI [0.11, 1.22]
6. Sensitivity analysis reduced heterogeneity from the considerable level (I^2^: 96%) to that might be not important level (I^2^=0%).
7. Wide CI, MD -56.95, 95% CI [101.59, -12.30] and MD -34.23, 95% CI [-44.87, -23.59] before and after sensitivity analysis, respectively
